# Pillararene-functionalised graphene nanomaterials

**DOI:** 10.1039/d0ra02964e

**Published:** 2020-05-14

**Authors:** Huacheng Zhang, Chao Li

**Affiliations:** School of Chemical Engineering and Technology, Xi'an Jiaotong University Xi'an Shaanxi 710049 China zhanghuacheng@xjtu.edu.cn; Department of Laboratory, Shandong University Hospital Jinan 250100 China lichao@sdu.edu.cn

## Abstract

Pillararene-modified graphene materials integrate the advantages of both graphene and pillararenes; *e.g.*, the cavity of pillararenes can recognise suitably sized electron-deficient and hydrophobic guest molecules *via* host–guest interactions, while the graphene composite is able to exhibit unique physiochemical properties including inertness, nanoscale, electrical and thermal structural properties. Those novel organic–inorganic hybrid composites can be efficiently prepared *via* both covalent and noncovalent bonds by classic organic reactions and supramolecular interactions, respectively. Pillararene-functionalised graphene materials have been used in various applications, such as electrochemical sensing guest molecules, performing as the platform for fluorescent probes, carrying out fluorescence quenching as the sensor, biosensing toxic molecules in cells, Raman and fluorescence bioimaging of cancer cells, photoacoustic and ultrasound imaging, as well as storage materials and reactors in energy fields.

## Abstract 2

The current research progress on diverse pillararene derivative functionalised graphene materials, including different synthesis strategies and various applications, is reviewed.

## Graphical abstract

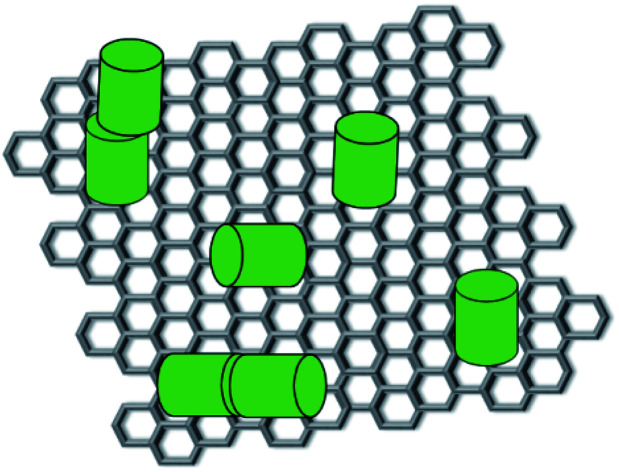

## Introduction

Graphene is a one-atom layered carbon material possessing two-dimensional (2D) planar structures with exceptional physiochemical properties and can be reliably produced in large quantities in a variety of forms including graphene oxide (GO), reduced graphene oxide (RGO) and exfoliated graphite.^1^ Due to its possession of particular electronic, mechanical and optical properties,^2^ as well as its guaranteed scalable production and supply, there has been tremendous interest in exploring the promising applications of graphene materials in wide areas of technologies and markets.^3,4^ In order to further improve performances and functions, the effective integration of graphene into hybrid composite materials has been well demonstrated to have scientific significance and practical applications.^3,5^

With the assistance of supramolecular recognition strategies as well as the nanotechnology approach,^6–10^ macrocycles with good aqueous solubility and recognition ability have been introduced to build graphene hybrids to improve their stability and dispersity in water,^11^ as well as expand their applications in bio-friendly and environmental areas such as biomedicines^3^ and clean energy.^2,5^

**Chart 1:**
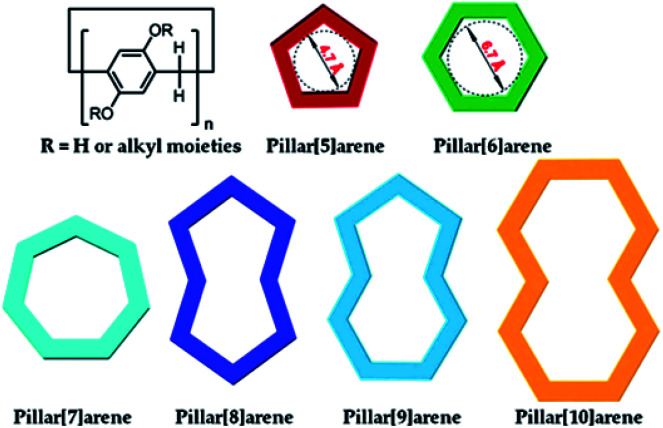
Structural and cartoon representations of typical pillar[*n*]arenes (*n* = 5–10). Reproduced from ref. 6 with permission from the American Chemical Society, Copyright 2015.

Pillar[*n*]arenes (Chart 1)^6,12–14^ possessing hydroquinone subunits linked by methylene bridges at *para* positions are new macrocyclic candidates for functionalising graphene materials, due to their high synthesis yields,^15^ efficient and easy modification,^8,14,16^ special electron-rich cavities for supramolecular recognition,^17,18^ rigid molecular scaffold, as well as unique planar chirality.^19–21^ In this review, we summarise current research progress on diverse pillararene derivative functionalised graphene materials by discussing their different synthesis strategies and various applications. We will try to answer a series of questions regarding pillararene-functionalised graphene materials, such as how to build these hybrids, what kind of driving forces could be used for the preparation procedure, how to confirm the structures of hybrid materials, *e.g.*, using which instruments, and which kind of unique properties from each composition will play the key role in potential practical applications.

**Scheme 1:**
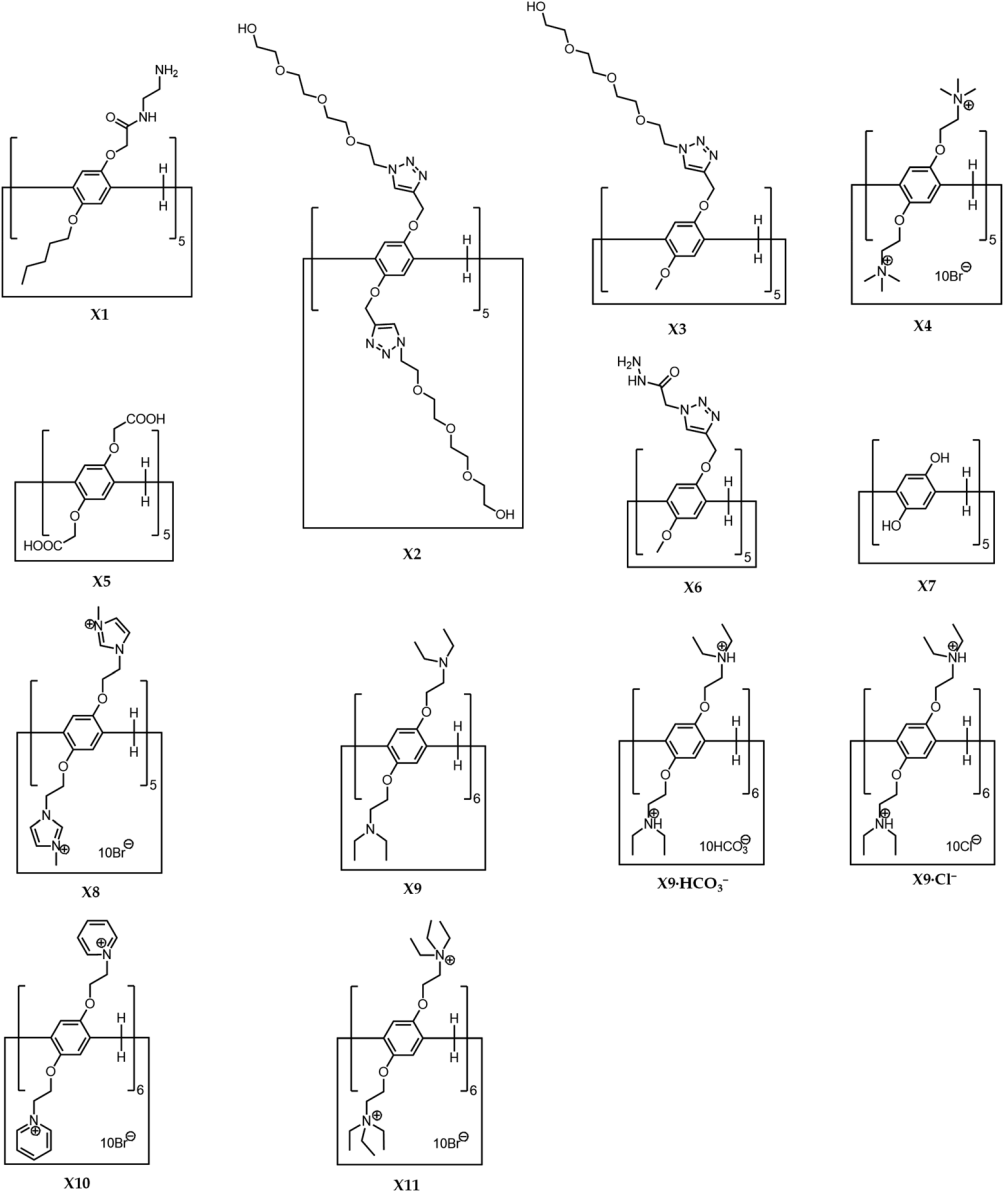
Structural illustration of pillararenes X1–X11.

The research area of pillararene-functionalised graphene nanomaterials is very new and under rapid development, and has attracted significant attention from related cross-field disciplines. The significance of working in this area is not in the development of new synthesis methods for hybrid materials but is focused on integrating two different functional organic/inorganic materials. We could summarise the progress based on different cases according to the timeline, but it might make the review confusing and boring by repeatedly exhibiting similar experimental details. Thus, we adopted a different strategy for organising this review, by separately analysing various synthesis strategies and applications. For example, due to the possession of active functional groups on the outer surface of graphene,^19,20^*e.g.*, hydroxyl and carboxylate moieties on GO, functionalised pillararene derivatives X1–X11 (Scheme 1) could be decorated onto graphene materials *via* covalent bonds by classic organic reactions *e.g.*, the EDC–NHS coupling reaction, as well as noncovalent bonds such as hydrogen bonding, electrostatic and π–π stacking interactions, leading to the formation of thermally stable organic–inorganic hybrid materials—pillararene-functionalised graphene materials. These hybrid materials integrate advantages from both graphene and pillararenes; for example, the cavity in the pillararene could contribute an electron-rich and hydrophobic environment to attract suitable guest molecules *via* supramolecular recognition, while the graphene composite exhibits its unique physiochemical properties such as inertness, nanoscale, electrical and thermal structural properties. As such, these integrated hybrid materials have wide practical applications in the electrochemical sensing of guest molecules, performing as the platform for fluorescent probes or carrying out fluorescence quenching as the sensor, biosensing toxic molecules in cells, Raman and fluorescence bioimaging of cancer cells, photoacoustic and ultrasound imaging, as well as acting as storage materials and reactors for energy fields. To help readers better understand the “history” of the research progress in this area, we provided Table 1, which is organised according to the timeline of different hybrid materials. It indicates the order of development of novel hybrid materials from diverse research groups. It also shows the changes in the research focused on this area such as adopting different driving forces for fabricating different hybrid materials. The cases as listed according to the timeline might appear in different categories in this review as shown in the preparation and application sections.

**Table 1:** Comparison of different hybrid materials fabricated by pillararenes, graphene and other compositions *via* diverse driving forces for various applications

Hybrid materials	Pillararenes	Graphene	Other compositions	Driving forces	Applications	Ref.
RGO–AP5–AuNPs	X1	RGO	Gold nanoparticles	Amido bond/π–π stacking interactions	Sensing	19
X2-GO/X3-GO	X2/X3	RGO	—	Hydrogen bonding	Biomedicines	20
MoS_2_/RGO(X4)	X4	RGO	MoS_2_	Electrostatic interactions	Lithium storage	22
RGO-CP5	X5	RGO	—	Ester bonds	Fluorescence sensing	23
HP-G	X6	RGO	—	Covalent bonds	Biosensor for Y1–Y5 with Y6 as the probe	24
RGO-P5A	X7	RGO	—	Covalent bonds	Sensing	25
MoS_2_/RGO-NP	X8	RGO	MoS_2_	Electrostatic interactions	Electrocatalyst	26
AmPA5-RGO	X1	RGO	—	π–π stacking and electrostatic interactions	Sensing	27
MoS_2_/RGO(X8)	X8	RGO		Electrostatic interactions	Lithium storage	28
GO@(H^+^˙X9)Y7	H^+^˙X9	GO	—	π–π stacking and host–guest interactions	Biomedicines	29
PCP6-RGO	X10	RGO	—	Electrostatic interactions	Sensing	30
CP5-RGO	X11	RGO	—	Electrostatic interactions	Sensing	31 and 32
(H^+^˙X9)@RGO	H^+^˙X9	RGO	—	Electrostatic interactions	Biomedicines	33

## Preparation of graphene materials decorated with functional pillararene derivatives

Functionalised pillararenes with diverse moieties can be loaded on the surface of graphene materials *via* covalent and noncovalent bonds. Covalent bonds are introduced mainly by classic organic reactions such as the EDC–NHS coupling reaction. Additionally, due to the possession of hydroxyl and carboxyl groups on the surface of graphene materials, supramolecular interactions such as hydrogen bonding and electrostatic interactions have been proved to be efficient and convenient in the fabrication of stable organic–inorganic hybrid materials. However, the current research efforts in this area have not performed the control experiment by comparing the advantages and disadvantages of covalent and noncovalent synthesis strategies. All the prepared hybrid materials as discussed here could further achieve diverse applications as indicated in the next section on applications.

**Fig. 1:**
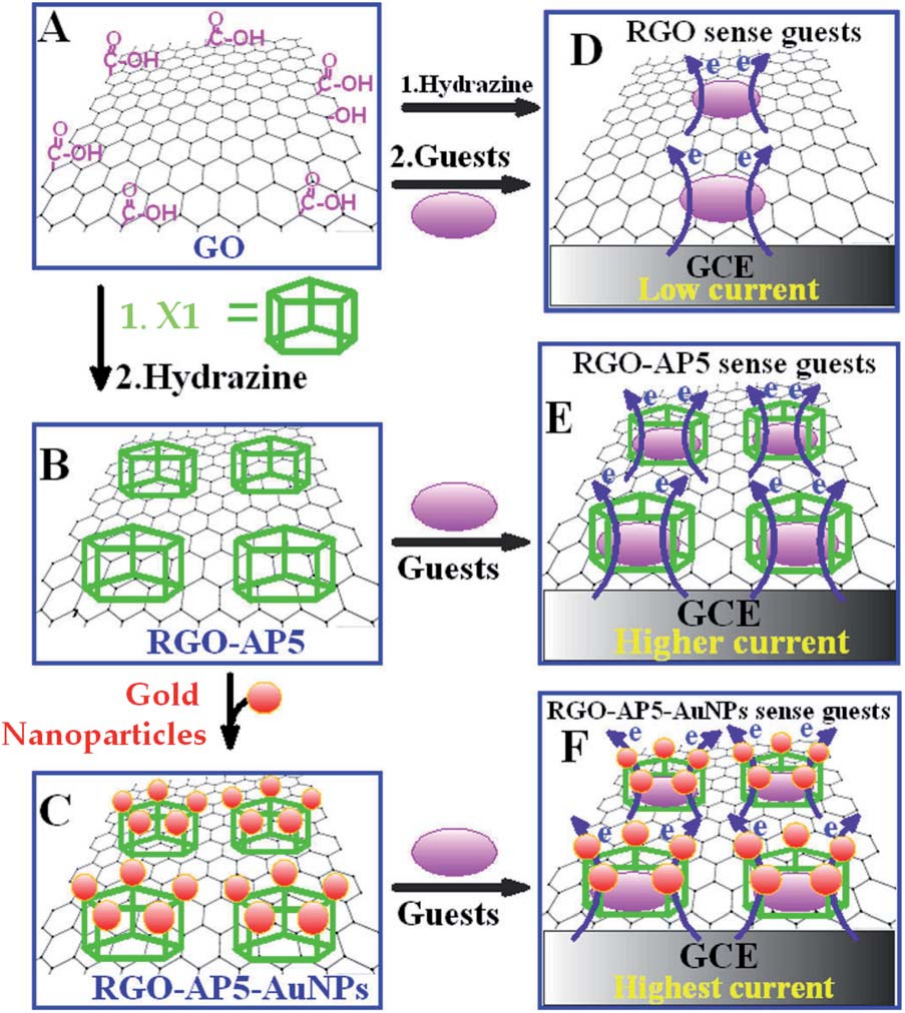
Proposed mechanisms for RGO, RGO–AP5 and RGO–AP5–AuNPs to sense target molecules, including dopamine, 4-acetamidophenol, uric acid, methylene blue, tryptophan and imidacloprid, as monitored by cyclic voltammetry. Reproduced from ref. 19 with permission from the American Chemical Society, Copyright 2013.

In the first pillararene-functionalised graphene material, the RGO was modified by amphiphilic pillar[5]arene X1 (Scheme 1)^19,34^ by successive preparation steps including assembling X1 on the surface of GO and reducing the mixture with hydrazine (Fig. 1). It is supposed that both covalent bonds, such as the amido bond, and noncovalent bonds, such as π–π stacking interactions, play significant roles to bridge pillararene units and RGO together (Table 1). The integration of X1 and RGO has further been fully characterised by FT-IR, UV-Vis spectra and thermal gravimetric analysis (TGA). Due to the large amount of X1 loaded on the surface of RGO, the fabricated hybrid nanomaterial RGO–AP5 could be much better dispersed in water as compared to the unmodified RGO. The gold nanoparticles were further uniformly assembled onto the outer surface of RGO–AP5*via* amido groups on X1, leading to the formation of ternary nanocomposites—RGO–AP5–AuNPs.

**Fig. 2:**
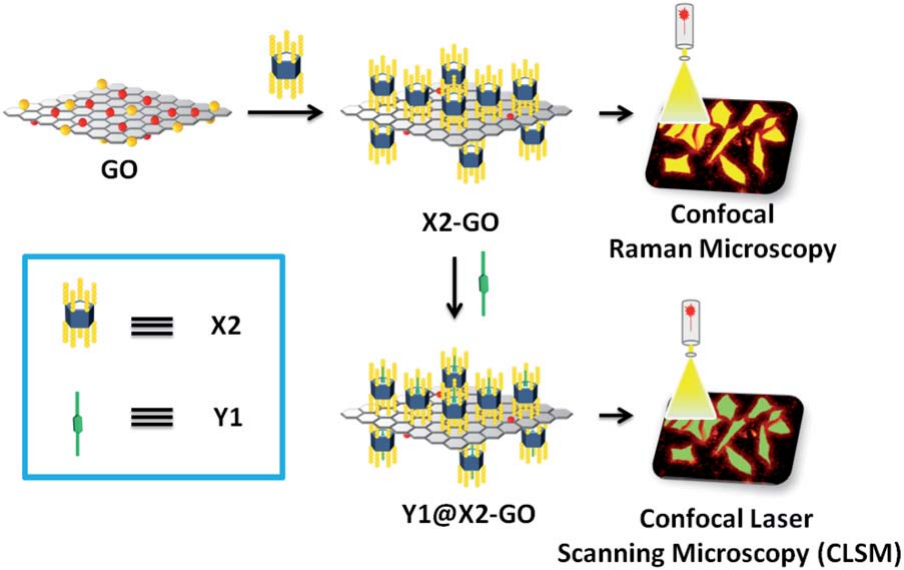
Illustration of water-soluble pillararenes decorated on the surface of graphene materials. Reproduced from ref. 20 with permission from Wiley-VCH GmbH & Co., Copyright 2014.

In another noncovalent functionalisation strategy, water-soluble pillar[5]arenes such as bola-amphiphilic X2 and tadpole-like amphiphilic X3 (Scheme 1)^20^ were loaded onto the surface of RGO *via* hydrogen-bonding interactions (Table 1). This was done by utilising a simple wet-chemistry strategy, *i.e.* treating an aqueous suspension of mixed GO and pillararenes with reducing agents such as hydrazine and ammonia, leading to a better and stable aqueous suspension of X2-GO and X3-GO (Fig. 2). The structures of the hybrid materials were further confirmed by TGA, FTIR, Raman spectra, transmission electron microscopy (TEM) and atomic force microscopy (AFM).

The classic organic synthesis methods were popular in the preparation of hybrid materials. For example, by using the EDC–NHS coupling reaction between carboxylate groups on X5 (Scheme 1) and hydroxy groups on the surface of graphene oxide sheets, as well as a similar follow-up chemical reduction with hydrazine hydrate,^23^ carboxylated pillar[5]arene (X5) was modified onto RGO *via* ester bonds, leading to the formation of RGO-CP5 with improved water-dispersibility. Similarly, the rim-differentiated functionalised pillar[5]arene X6 (Scheme 1)^24^ was grafted onto graphene *via* the EDC–NHS coupling reaction between carboxyl groups on graphene and hydrazine subunits on pillararenes, leading to the formation of HP-G composites as confirmed by AFM, FT-IR, X-ray photoelectron spectroscopy (XPS) and fluorescence spectroscopy.

Alternatively, per-hydroxylated pillar[5]arene (X7, Scheme 1) could covalently decorate graphene, as confirmed by FT-IR, UV-Vis spectra, TGA and TEM, through a particular procedure including the treatment of a mixture of graphene oxide and X7 with thionyl chloride, coating the obtained hybrid materials on the surface of the glassy carbon electrode, as well as irradiating the decorated glassy carbon electrode under the Xe lamp.^25^

Pillar[5]arenes can also perform as a bridge to assist in the fabrication of advanced hybrid composites. For example, the composites containing MoS_2_ and RGO were prepared by a hydrothermal reduction between Na_2_MoO_4_ and l-cysteine (l-Cys) in the presence of graphene oxide sheets.^22^ The positively charged trimethylammonium perfunctionalised pillar[5]arene (X4, Scheme 1) was introduced during the synthesis procedure to modify the graphene oxide sheets *via* electrostatic interactions (Table 1). Due to their possessing rigid chemical structures and multivalent noncovalent interactions with hybrid materials, cationic pillar[5]arene amphiphiles X4 affect the microstructure and electrochemical performances of MoS_2_/RGO composites, leading to the formation of irregular pores and apertures in MoS_2_/RGO composites with few-layer MoS_2_ sheets well dispersed and anchored on the surface of RGO. As confirmed by TEM and scanning electronic microscopy (SEM), MoS_2_/RGO has the morphology of wrinkled thin flakes. In another example, by using a similar hydrothermal synthetic route and driving forces for the fabrication of hybrid materials, water-soluble *N*-methylimidazole perfunctionalised pillar[5]arene X8 (Scheme 1) also assisted in the construction of MoS_2_/RGO-NP as the mediator.^26,28^ Amphiphilic X1 could also be grafted onto the surface of RGO *via* electrostatic and π–π stacking interactions by adjusting the pH of aqueous suspensions, leading to the formation of amPA5-RGO.^27^ Pyridium perfunctionalised pillar[6]arene X10 (Scheme 1)^30^ and tertiary ammonium perfunctionalised pillar[5]arene X11 (Scheme 1)^31,32^ could also be loaded on the surface of graphene *via* electrostatic interactions and result in the formation of hybrid materials PCP6-RGO and CP5-RGO, respectively.

## Application of pillararene-functionalised graphene materials

Pillararene-functionalised graphene materials have been used in various applications including (1) electrochemical and fluorescence sensing, (2) biomedicines and biosensing, Raman and fluorescent bioimaging, as well as photoacoustic and ultrasound imaging, (3) lithium storage and catalysis.

### Sensing

In their application to electrochemical sensing, the electrical properties of graphene materials allow them to generate significant electrical signals towards guest molecules introduced by the supramolecular recognitions of pillararene cavities. In their application in fluorescence sensing, the two-dimensional morphologies of graphene materials perform as the platform to load pillararenes on the surface, which further serve as the active recognition sites for fluorescent guests or probes.

The electrochemical sensing of guest molecules is carried out by coating nanocomposites onto the glassy carbon electrode (GCE) by cyclic voltammetry. For example, the hybrid nanocomposites RGO–AP5 and RGO–AP5–AuNPs have selective supramolecular recognition and the capacity for enrichment towards guest molecules, as analysed among electroactive candidates such as dopamine, 4-acetamidophenol, uric acid, methylene blue, tryptophan and imidacloprid.^19^ Particularly, ternary hybrid materials RGO–AP5–AuNPs exhibited the best sensing abilities towards dopamine with a broad linear range from 1.5 × 10^−8^ to 1.9 × 10^−5^ mol L^−1^, as well as a low detection limit of 1.2 × 10^−8^ mol L^−1^ at a signal-to-noise ratio of 3, due to the synergetic action of multifunctional properties such as the capacity for supramolecular recognition by pillararenes, catalytic properties of gold nanoparticles as well as the electrochemical properties of graphene (Fig. 1).

In another example, the hybrid composites RGO-P5A^25^ have a good capacity for recognising guest molecules such as dopamine, ascorbic acid and uric acid, and exhibit selective electrochemical response towards dopamine, due to the molecular recognition by the cavities of pillararenes. Pillararenes also perform as promotors for enhancing the electrochemical response. In comparison with cyclodextrin-modified RGO, CP5-RGO^32^ exhibit better electrochemical catalytic activity as a sensor, *e.g.*, the trace detection of methyl parathion with the low detection limit of 3.0 × 10^−10^ mol L^−1^ (S/N = 3) and the linear response range of 1.0 × 10^−9^ to 1.5 × 10^−4^ mol L^−1^.

Pillararene-functionalised graphene materials could also be applied in fluorescent dye sensing. For example, due to the carboxylate perfunctionalised pillar[5]arenes (X5), the composite RGO-CP5 nanosheets exhibit enhanced fluorescence-quenching properties towards rhodamine 6G and neutral red in comparison to unmodified RGO,^23^ due to the π–π stacking interactions between aromatic dyes and RGO, as well as possible host–guest interactions between dyes and the cavity of pillar[5]arenes.

Due to the difference in supramolecular recognition towards guest molecules such as acridine orange and acetaminophen *via* hydrophobic interactions by the cavity of pillar[5]arene, the AmPA5-RGO composite can act as a fluorescent sensing platform for the detection of acetaminophen, in which the acridine orange performed as the signal probe.^27^ It has been proved that this fluorescence sensing system has a low detection limit of 5.0 × 10^−8^ mol L^−1^ with a S/N ratio of 3, as well as a linear response range of 1.0 × 10^−7^ to 4.0 × 10^−6^ and 4.0 × 10^−6^ to 3.2 × 10^−5^ mol L^−1^.

By using a similar fabrication strategy,^30^ PCP6-RGO also performs as a selective and sensitive fluorescence platform for detecting trinitrophenol with acridine orange as the probe, exhibiting a low detection limit of 3.5 × 10^−9^ mol L^−1^ (S/N = 3) and a wider linear response of 1.0 × 10^−8^ to 5.0 × 10^−6^ and 5.0 × 10^−6^ to 1.25 × 10^−3^ mol L^−1^.

### Biomedicines

The introduction of modified pillararene derivatives can not only improve the aqueous solubility of graphene materials, but can also enhance the biocompatibility of hybrid materials. Furthermore, pillararenes donate their electron-rich cavities for the supramolecular recognition of electron-deficient guests, which provides the possibility of including and delivering particular guest molecules for biomedicines. The unique physiochemical properties of graphene materials have been primarily explored, such as thermal structural properties, *e.g.*, Raman imaging or irradiating under near-infrared (NIR) light.

**Fig. 3:**
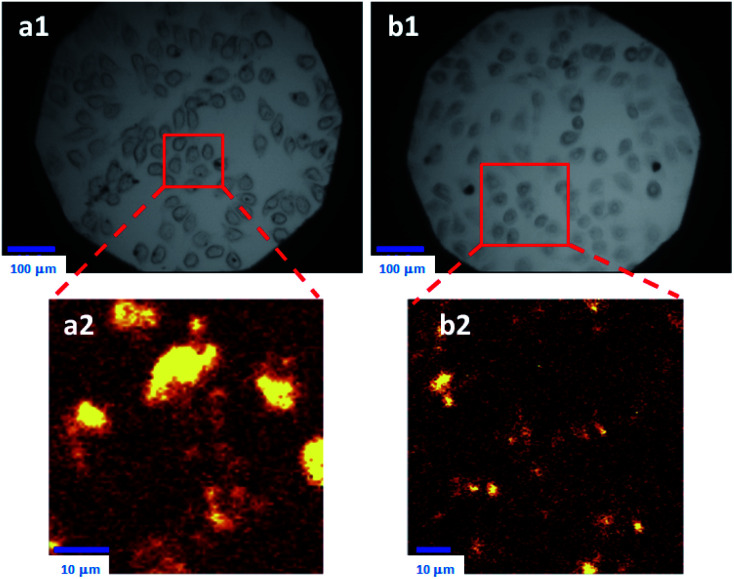
Images taken by confocal Raman microscope for HeLa cells: (Top) after treatment with X2-GO (a1) and X3-GO (b1) for 24 h, bright field. (Bottom) Enlarged areas as detected by Raman spectra (a2 and b2). Reproduced from ref. 20 with permission from Wiley-VCH GmbH & Co., Copyright 2014.

**Fig. 4:**
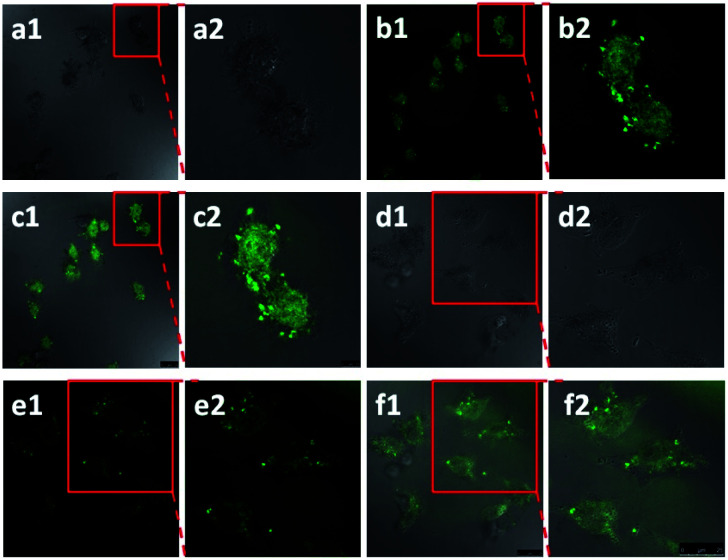
Confocal laser scanning microscope (CLSM) images of HeLa cells after treatment with the X2-GO Y1 inclusions for 24 h in (a1) bright field, (b1) green field and (c1) merged image of (a1) and (b1); the enlarged area as shown in (a2) bright field, (b2) green field and (c2) merged image of (a2) and (b2). CLSM images of HeLa cells after being treated with X3-GO Y1 inclusions for 24 h as the control experiment in (d1) bright field, (e1) green field and (f1) merged image of (d1) and (e1); the enlarged area as shown in (d2) bright field, (e2) green field and (f2) merged image of (d2) and (e2). Reproduced from ref. 20 with permission from Wiley-VCH GmbH & Co., Copyright 2014.

**Scheme 2:**
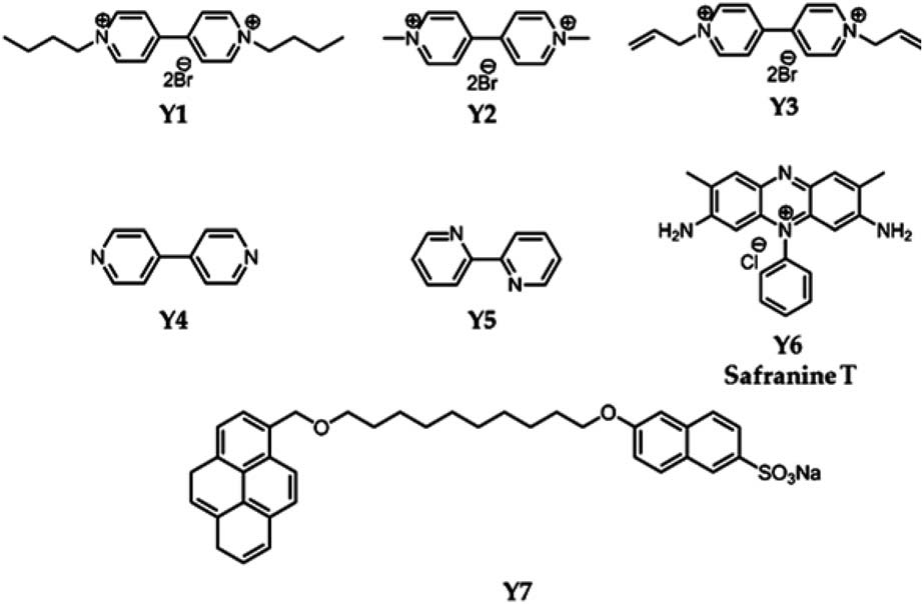
Structural illustration of guest and other significant molecules Y1–Y7.

Functionalised pillararenes having good water solubility could perform as stabilizers for graphene materials in aqueous solution for biological applications, as well as contribute their cavities for hosting suitable guest molecules *via* supramolecular recognition. For example, after integrating with water-soluble pillararenes, hybrid materials X2-GO and X3-GO (Fig. 2) not only exhibited improved aqueous suspensions, but also had good biocompatibility as confirmed by the 3-(4,5-dimethylthiazol-2-yl)-2,5-diphenyltetrazolium bromide (MTT) assay.^20^ The X2-GO and X3-GO nanocomposites were further endocytosed by HeLa cells for dual-mode Raman (Fig. 3) and fluorescence bioimaging (Fig. 4) *in vitro*. It was found that the bola-amphiphile X2-functionalised hybrid materials performed better in both Raman and fluorescence imaging as compared to the tadpole-like amphiphile X3-functionalised ones. In the study of fluorescence imaging, the electron-deficient bipyridinium derivative Y1 (Scheme 2) was employed as the model fluorescent dye, which could be included in the electron-rich cavity of water-soluble pillar[5]arene-derivatives.

**Fig. 5:**
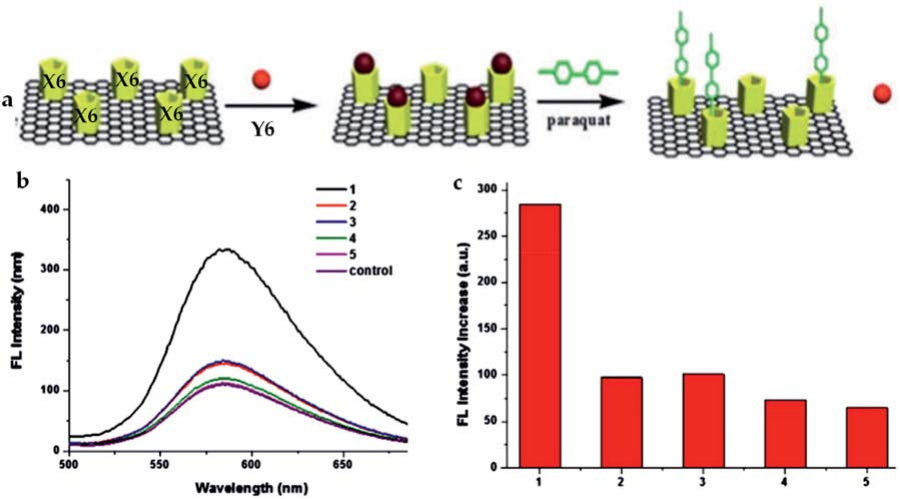
Structural illustration of safranine T as well as the process during which the fluorescent indicator-displacement assay was employed to accomplish the fluorescence “off-on” mechanism for the detection of Y2 (a). Fluorescence spectra of the probe containing HP-G/safranine T in the presence of Y1–Y5 (b). Relative fluorescence intensity ((c), *I*/*I*_0_) of Y1–Y5, as well as *I*_0_ and *I*, indicating the fluorescence intensity without and with the paraquat, respectively. Reproduced from ref. 24 with permission from The Royal Society of Chemistry, Copyright 2016.

**Fig. 6:**
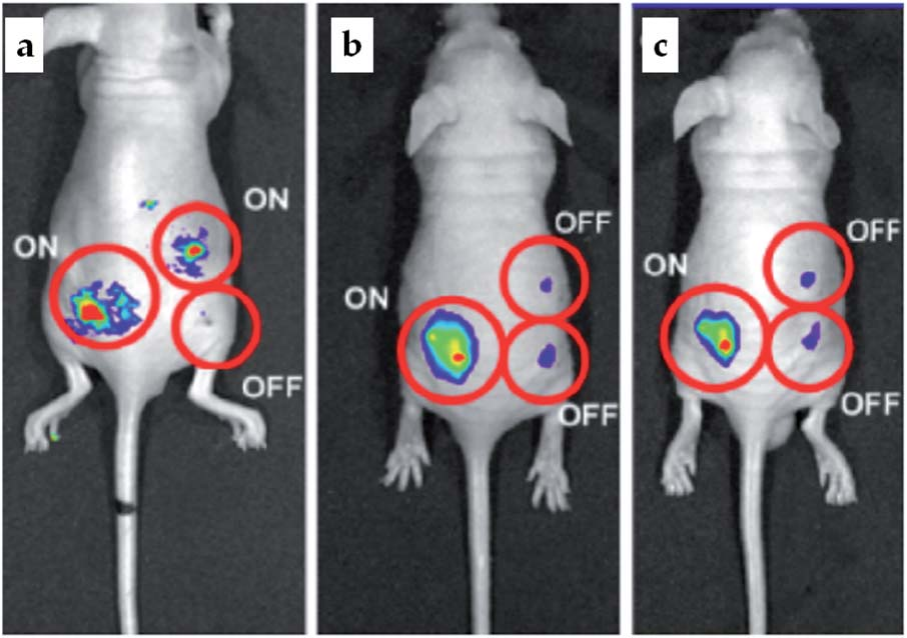
*In vivo* release of safranine T from the composites of HP-G in the presence and absence of Y2 (a). The fluorescence of safranine T is “ON” in the absence of HP-G composites (left), “OFF” in the presence of HP-G composites (bottom, right), and “ON” again in the presence of both HP-G and Y2 after the procedure of fluorescence recovery (up, right). Similarly, after treatment with Y3 (b) and Y1 (c) by using the HP-G/safranine T probe, the fluorescence was still “OFF”, indicating no recovery (top, right). Reproduced from ref. 24 with permission from The Royal Society of Chemistry, Copyright 2016.

Pillararene-functionalised graphene materials could perform as a platform for fabricating biosensors in biological samples, during which pillararenes contribute to supramolecular recognition *via* host–guest interactions with targeted analytes. For example, the pillar[5]arene X6-functionalised graphene HP-G could perform as a fluorescent probe together with safranine T (Y6, Scheme 2) for paraquat (Y2, Scheme 2) among different paraquat analogues (Y1–Y5, Scheme 2) in living cells and mice *via* selective host–guest interactions.^24^ The fluorescent indicator-displacement assay was employed during this process as a practical strategy by utilising safranine T as the indicator (Fig. 5), and graphene could translate weak interactions into a sensitive fluorescence signal. Furthermore, after treatment with Y2 (Fig. 6a), the total fluorescence intensities were enhanced, indicating the release of dye molecules from the probe of HP-G/safranine T in mice. After treatment with Y3 and Y1, the fluorescence remained “OFF”, indicating that the probe was not responsive towards these analogues of paraquats (Fig. 6b and c). In another example, (H^+^˙X9)@RGO^33^ (Table 1) performs as the fluorescent sensing platform for detecting insulin, a peptide hormone synthesised in the cells of the islet of Langerhans, with rhodamine B as the probe, also based on the competitive recognition of cationic pillar[6]arene X9 with the low detection limit of 3.0 × 10^−9^ mol L^−1^ and a linear response range of 1.0 × 10^−8^ to 5.0 × 10^−7^ and 1.0 × 10^−3^ to 1.6 × 10^−2^ mol L^−1^.

**Fig. 7:**
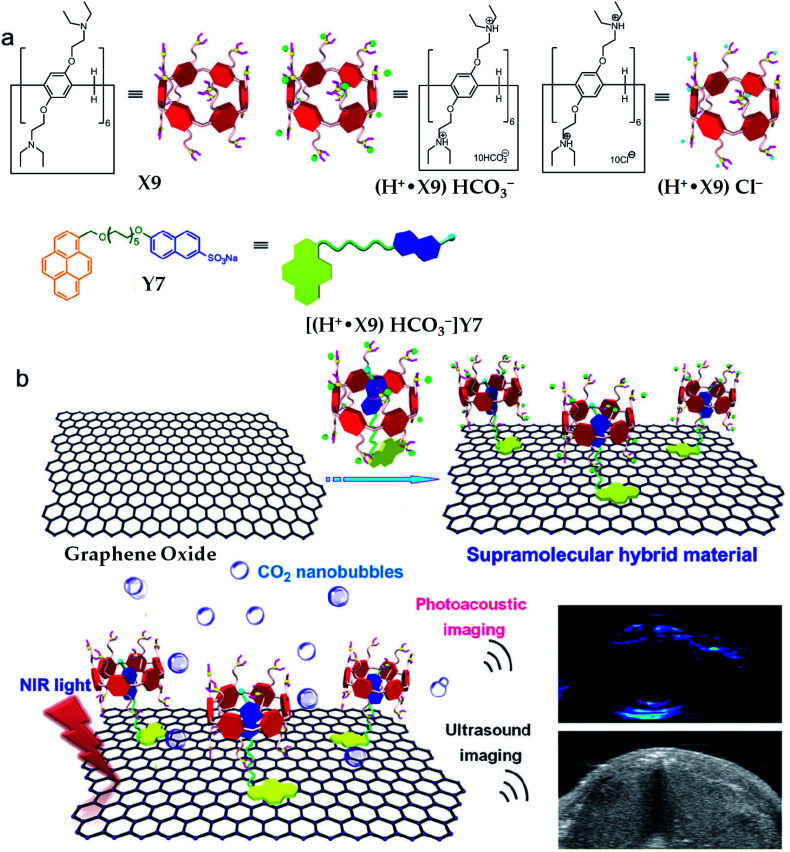
Illustration of the synthesis route for GO@(H^+^˙X9)Y7, which can be further used for NIR light-triggered photoacoustic and ultrasound imaging. Reproduced from ref. 29 with permission from The Royal Society of Chemistry, Copyright 2018.

The water-soluble tertiary amine perfunctionalised pillar[6]arene (X9, Scheme 1)-modified graphene hybrid materials GO@(H^+^˙X9)Y7 (Table 1)^29^*via* host–guest as well as π–π stacking interactions could employ the NIR light-mediated photothermal effect of graphene oxide to make the bicarbonate anions on the surface of the hybrid materials decompose into carbon dioxide (CO_2_) nanobubbles under the irradiation of a NIR laser (Fig. 7). Due to the small size and good tissue permeability, the obtained CO_2_ nanobubbles were further applied to enhance the photoacoustic and ultrasound imaging as confirmed by *in vitro* and *in vivo* investigations. In this hybrid material, the CO_2_-responsive supramolecular complexes (H^+^˙X9)Y7 performed significant roles in increasing the NIR absorption as monitored by the changes in temperature shown by an IR camera.

### Lithium storage and catalysis

**Fig. 8:**
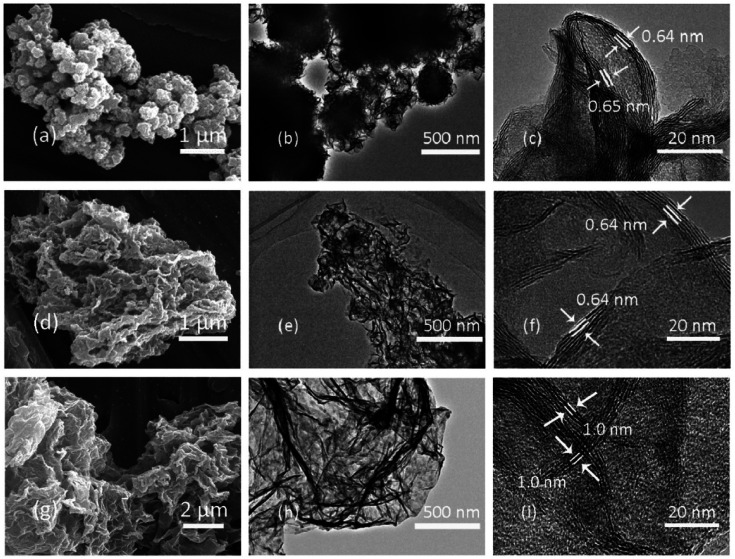
Images as observed by SEM and TEM for pristine MoS_2_ (a–c), MoS_2_/RGO (d–f) and MoS_2_/RGO-NP (g–i). Reproduced from ref. 26 with permission from The Royal Society of Chemistry, Copyright 2017.

Amphiphilic pillararenes are a significant bridge during the connection of diverse hybrid materials. For example, with the assistance of cationic amphiphilic pillararenes as the mediators, hybrid materials could be fabricated by connecting MoS_2_ and RGO together. During the preparation, the positively charged pillar[5]arene X8 could mitigate the charge incompatibility between MoO_4_^2−^ and the graphene oxide sheets in the fabrication of the hybrid composites MoS_2_/RGO-NP, in which the MoS_2_ sheets were well dispersed on the surface of RGO containing exposed active edge sites (Fig. 8).^26^ The obtained hybrid materials MoS_2_/RGO^22^ could be applied to lithium storage as well as anode materials for lithium-ion batteries; *i.e.* MoS_2_/RGO has a specific capacity of 1050–1140 mA h g^−1^ with good cyclic performance.^22^ Particularly, due to their possessing robust composite structures and better synergic effects between few-layer MoS_2_ and RGO sheets, these kinds of composites have an enhanced high-rate capacity of 815–875 mA h g^−1^ at a current density of 1000 mA g^−1^ as compared to pristine MoS_2_. In another example, MoS_2_/RGO prepared by employing a different positively charged mediator (X8)^28^ could deliver a reversible specific capacity as high as 1289 mA h g^−1^ at the same current density of 1000 mA g^−1^. Particularly, the reversible capacity of 996 mA h g^−1^ can be retained after 1100 cycles at unchanged current density. Furthermore, the hydrogen evolution reaction is the key step in producing hydrogen *via* electrochemical water splitting using solar energy or electricity. The MoS_2_/RGO-NP composites (Fig. 8)^26^ could achieve not only improved hybridisation, but also good electrocatalytic performance for the hydrogen evolution reaction with a low Tafel slope of 44.5 mV dec^−1^, which is the intrinsic property of an electrocatalyst.

## Overview and outlook

Recent progress in the synthesis and application of pillararene-functionalised graphene materials is summarised in this review. Both covalent and noncovalent bonds were used to load pillararenes onto the outer surface of nanoscale graphene materials. After the introduction of pillararenes, supramolecular recognition can be employed in the hybrid composites for recognising interesting guest molecules through host–guest interactions. Due to the electrical properties of graphene, pillararene-functionalised graphene materials could be used for the electrochemical sensing of particular guest molecules that are suitable for the electron-rich pillararene cavities. If the guest molecules have fluorescence or act as the probe, the hybrid composites containing pillararene-functionalised graphene could further perform as the platform for fluorescence sensing. Additionally, the hybrid materials could integrate the properties of functionalised pillararenes, *e.g.*, recognising specialised bioimaging reagents, as well as the unique physiochemical properties of graphene, *e.g.*, thermal structural properties together, leading to applications of biomedicines such as biosensing, Raman and fluorescence bioimaging, as well as photoacoustic and ultrasound imaging. Furthermore, amphiphilic pillararenes could serve as a bridge to decorate graphene materials with other inorganic materials *via* supramolecular interactions, resulting in more interesting applications in energy fields such as in lithium storage and catalysis.

A lot of perspective work in this area is still attractive for researchers from different fields such as synthesis, mechanism studies and material sciences.

(1) Regarding organic components, *i.e.* pillararenes, only pillar[5]arenes and pillar[6]arenes are employed in the fabrication of current graphene hybrid materials, and diverse-sized pillararenes^35^ might be introduced into the hybrid materials in future work for various types of supramolecular recognition as well as more interesting applications based on those larger cavities.

(2) Regarding the inorganic components, new synthesis strategies such as the strategy of covalent cross-linking^36^ should be borrowed to expand the current categorization and morphology of 2D layered inorganic structures, which could provide the possibility for applying the obtained hybrid materials to, *e.g.*, the hydrogen evolution reaction.^37,38^ Furthermore, the special physiochemical functions of graphene, including but not limited to nanoscale morphologies, as well as inert, thermal structural, optical and electrical properties should be deeply explored in these hybrid materials.^39^

(3) Regarding the study of the binding mechanism, the driving forces during the fabrication of pillararene-functionalised graphene materials need further investigations such as computational calculations, high-resolution electron microscopic observations as well as crystallographic studies. The different synthesis strategies regarding covalent and noncovalent interactions should be re-evaluated to ascertain their advantages and disadvantages.

(4) Concerning the expansion of the application fields, more functional groups could be introduced into the hybrid composites *via* either the modification of pillararene derivatives or the decoration of the outer surface of graphene materials. The introduction of various functional moieties^40,41^ could allow pillararene-functionalised graphene materials to, for example, have better water dispersity and solubility, which promote the materials to have better biocompatibility and lower cytotoxicity, leading to the expansion of application to more aspects of biomedicines such as diagnosis and combined therapy.^42^

## Abbreviation

2D: Two-dimensional
AFM: Atomic force microscopy
CLSM: Confocal laser scanning microscopy
GCE: Glassy carbon electrode
GO: Graphene oxide
l-Cys: l-Cysteine
MTT: 3-(4,5-Dimethylthiazol-2-yl)-2,5-diphenyltetrazolium bromide
NIR: Near-infrared
RGO: Reduced graphene oxide
SEM: Scanning electronic microscopy
TEM: Transmission electron microscopy
TGA: Thermal gravimetric analysis
XPS: X-ray photo-electron spectroscopy

## Conflicts of interest

The authors declare no conflict of interest.

## Supplementary Material
